# Exploratory RNA Sequencing Reveals Systemic Metabolic Dysregulation in Alzheimer’s Disease: Insights from a Diverse Latin American Cohort

**DOI:** 10.1007/s12035-025-05597-8

**Published:** 2026-01-27

**Authors:** Lina M. Villegas-Trujillo, Beatriz Parra, Diana López-Álvarez, Lina M. Gonzalez-Ojeda, Alejandra Torres-Parga, Sebastián Cardona, Nelson Rivera-Franco, Juan F. Cardona

**Affiliations:** 1 PhD Program in Biomedical Sciences, School of Basic Sciences, Faculty of Health, Universidad del Valle, Cali, Colombia; 2 School of Basic Sciences, Department of Microbiology, Faculty of Health, Universidad del Valle, Cali, Colombia; 3 Department of Biological Sciences, Faculty of Agricultural Sciences, Universidad Nacional de Colombia, Palmira, Colombia; 4 PhD Program in Psychology, Faculty of Psychology, Universidad del Valle, Cali, Colombia; 5 Hospital Universitario del Valle “Evaristo Garcia” E.S.E., Cali, Colombia; 6 Neurology Residency Program, Department of Internal Medicine, Faculty of Health, Universidad del Valle, Cali, Colombia; 7 Department of Biology, Faculty of Natural and Exact Sciences, Universidad del Valle, Cali, Colombia; 8 Department of Developmental Science, Cognition and Neuroscience, Faculty of Psychology, Universidad del Valle, Calle 13 No. 100-00, Edificio 388 Oficina 4035, Cali, Colombia

**Keywords:** Alzheimer’s disease, Cognitive dysfunction, Gene expression profiling, RNA-Seq

## Abstract

Alzheimer’s disease (AD) is characterized by an insidious onset and complex pathophysiology, necessitating the development of effective strategies for early detection and intervention. This exploratory study aimed to identify differentially expressed genes (DEGs) and disrupted molecular pathways in AD by analyzing blood samples from participants recruited in Valle del Cauca, Colombia, a region with high genetic admixture and persistent underrepresentation in genomic research. A total of 41 individuals (AD, *n* = 14; cognitively healthy controls (CHC), *n* = 27) were included. Groups did not differ significantly in age, education, sex distribution, or vascular comorbidities. Peripheral blood RNA was sequenced using 150-bp paired-end reads, and transcriptomic profiling revealed 399 DEGs, with 378 upregulated and 21 downregulated in the AD group. Key genes such as APOE, MMP2, PPARG, and TUBB3 were enriched in the Metabolism of Proteins pathway. At the same time, TUBB3, CACNA2D1, and GABBR2 were implicated in transmission across chemical synapses, suggesting synaptic signaling and protein metabolism dysregulation. Multiple factor analysis (MFA), integrating gene expression with neurocognitive and functional outcomes, revealed distinct molecular signatures associated with cognitive decline and functional impairment. These findings highlight the role of systemic metabolic dysfunction and synaptic dysregulation in AD pathogenesis. By focusing on an ancestrally diverse cohort, this study underscores the critical need to expand the molecular characterization of AD beyond European-ancestry populations, informing the development of inclusive biomarkers and precision strategies for early diagnosis and intervention.

**Graphical Abstract:**

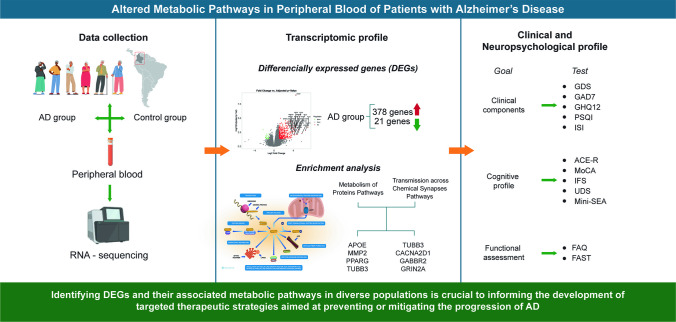

**Supplementary Information:**

The online version contains supplementary material available at 10.1007/s12035-025-05597-8.

## Introduction

Alzheimer’s disease (AD) is a complex and heterogeneous neurodegenerative disorder shaped by the interplay of genetic, epigenetic, and environmental factors, challenging the traditional monogenic paradigm of neurodegenerative disease [1–4]. Its insidious onset and multifactorial pathophysiology highlight the urgent need for early detection strategies. In this context, molecular biomarkers are gaining traction for diagnosis, staging, and therapeutic monitoring [5]. However, standard diagnostic approaches, clinical evaluations, neuropsychological assessments, neuroimaging, and cerebrospinal fluid (CSF) analysis often present logistical, financial, and cultural challenges. In particular, procedures such as CSF collection are invasive and not always feasible in routine clinical settings, especially in resource-limited regions [6, 7]. While widely used, neuropsychological testing may be influenced by language, literacy, and sociocultural background, limiting its generalizability in low- and middle-income countries (LMICs) [8].

These limitations underscore the need for scalable, non-invasive biomarkers that can support earlier detection, especially in health systems where infrastructure or access to specialized services is limited. Importantly, molecular biomarkers are not intended to replace established diagnostic protocols but rather to complement them, enhancing precision, interpretive value, and reach in under-resourced contexts.

Recent advances in high-throughput sequencing technologies have enabled the discovery of blood-based RNA signatures as promising diagnostic candidates [9, 10]. Peripheral blood offers a minimally invasive, easily accessible biological matrix that reflects systemic processes, including inflammation, metabolic dysfunction, and stress responses, features increasingly recognized in AD pathogenesis [11]. Transcriptomic profiling of whole blood has been shown to detect disease-relevant molecular patterns and holds potential for early detection, stratification, and personalized intervention planning [12]. However, most transcriptomic biomarker studies have been conducted in high-income countries, with samples primarily drawn from populations of European ancestry, which limits their external validity [13–15].

This underrepresentation is particularly problematic in regions such as Latin America, where the intersection of structural inequality, environmental heterogeneity, and genetic admixture uniquely shapes health outcomes [16–18]. In Colombia, centuries of admixture among Indigenous, African, and European groups have produced high interindividual and regional genetic diversity [19, 20]. These ancestry-related patterns are not only of anthropological interest but have critical implications for gene expression, immune function, and disease susceptibility. However, they remain largely unaccounted for in current AD biomarker research.

The southwestern department of Valle del Cauca, home to the city of Cali and its surrounding regions, exemplifies these dynamics. Shaped by pre-Columbian settlement, colonial trade, and post-independence migration, it is among the most ancestrally diverse regions in the Americas. However, it remains remarkably underrepresented in genomic studies of neurodegenerative diseases, including AD [21]. While Colombia has contributed key insights into familial early-onset AD through the PSEN1 E280A mutation identified in Antioquia [22–24], this mutation accounts for a small and geographically concentrated subset of the global AD burden. In contrast, late-onset, sporadic AD, which is shaped by polygenic risk, social determinants, and limited access to healthcare, represents the most prevalent form worldwide [25–27].

Despite this, ancestrally diverse populations, particularly in regions affected by historical marginalization, remain underrepresented in genomic datasets, limiting our ability to build inclusive, precision-based diagnostic strategies [16]. Nowhere is this inequity more evident than in Colombia’s Pacific region, where African and Indigenous ancestries are overrepresented, and where social exclusion has long restricted participation in biomedical research. Their systematic absence from transcriptomic analyses of AD perpetuates knowledge gaps and diagnostic disparities in neurodegenerative medicine [13, 21].

To address this gap, the present study used high-throughput RNA sequencing and systems biology tools to identify differentially expressed genes (DEGs) in peripheral blood samples from individuals with AD. We integrated transcriptomic data with neurocognitive and functional assessments to explore clinically relevant molecular signatures. While exploratory in scope, our findings contribute to a growing body of research calling for biomarker development that reflects the global diversity of AD presentation and supports rather than replaces current clinical diagnostic frameworks.

## Materials and Methods

### Participants and Study Setting

This study was conducted in Santiago de Cali, Colombia, a region with high levels of genetic admixture and historical underrepresentation in genomic research. Participants were recruited from the Centro Médico de Atención Neurológica “Neurólogos de Occidente,” which provides outpatient services to socioeconomically diverse populations in the southwest of the country. The cohort included individuals clinically diagnosed with AD and cognitively healthy controls (CHC). Control participants were matched to AD cases by age and sex and recruited through a public call in Cali.

### Inclusion Criteria and Diagnostic Framework

AD diagnoses were established by consensus among neurologists, neuropsychologists, and general practitioners involved in the study. In cases of diagnostic uncertainty, a neuropsychiatric evaluation was sought to confirm eligibility. All diagnoses adhered to the National Institute of Neurological and Communicative Disorders and Stroke–Alzheimer’s Disease and Related Disorders Association (NINCDS–ADRDA) criteria, consistent with NIA-AA recommendations [28, 29]. Beyond comprehensive neuropsychological and functional assessments, all participants underwent 3 T MRI scans, which were systematically reviewed to identify disease-related atrophy and to exclude other causes of dementia, including significant cerebrovascular disease, such as large infarcts, Fazekas grade 3 white matter lesions, mass lesions, or alternative neurodegenerative syndromes. Biomarkers from CSF or PET/SPECT were not collected due to feasibility constraints. Inclusion criteria for all participants required the absence of acute medical illness, cancer, or recent hospitalization. All participants provided written informed consent before enrollment.

### Clinical and Neurocognitive Assessment

Demographic, clinical, and neuropsychological assessments were conducted to characterize participants’ cognitive status and health background comprehensively. Variables including age, sex, years of education, handedness, socioeconomic status, and vascular comorbidities (e.g., arrhythmia, cardiac surgery, diabetes, dyslipidemia, hypertension, hypothyroidism, and obesity) were collected through structured interviews and a review of medical charts.

Global cognitive functioning was assessed using the Addenbrooke’s Cognitive Examination–Revised (ACE-R) and the Montreal Cognitive Assessment (MoCA), both widely validated tools in clinical and research settings. Executive functioning was evaluated using the INECO Frontal Screening (IFS), which captures deficits in inhibitory control, abstraction, and working memory. Education-adjusted norms were applied to minimize the influence of educational disparities, which are critical in LMIC settings where formal schooling is often limited or inconsistent.

Domain-specific cognitive abilities were further characterized using the Uniform Data Set (UDS) neuropsychological battery from the US National Alzheimer’s Coordinating Center (NACC), providing standardized composite scores for episodic memory, language, and visuospatial function. The Mini-Social Cognition and Emotional Assessment (Mini-SEA) was administered to assess socioemotional cognition, offering sensitivity to early changes in theory of mind and affective processing in neurodegenerative diseases.

Functional status was evaluated using the Functional Activities Questionnaire (FAQ) and the Functional Assessment Staging Tool (FAST), capturing declines in instrumental and basic activities of daily living, respectively. Emotional and psychiatric symptoms were assessed using the Geriatric Depression Scale (GDS) and the Generalized Anxiety Disorder scale (GAD-7). Sleep quality and related disturbances were measured with the Pittsburgh Sleep Quality Index (PSQI), the General Health Questionnaire (GHQ-12), and the Insomnia Severity Index (ISI), given the growing evidence linking sleep disruption to neurodegeneration.

All assessments were performed by trained clinicians or graduate-level researchers under supervision, ensuring consistency and inter-rater reliability. These evaluations provided a multidimensional phenotypic profile, essential for subsequent correlation with gene expression patterns.

### RNA Extraction and Quality Assessment

Peripheral blood samples were collected in the morning, under fasting conditions and without tourniquet application, to minimize circadian and hemodynamic variability. Venipuncture was performed using PAXgene Blood RNA tubes (Becton Dickinson, Franklin Lakes, NJ), following the manufacturer’s protocol to preserve RNA integrity. All samples were immediately placed on dry ice and transported to the Molecular Biology Laboratory at Universidad del Valle within 2 h for processing.

Total RNA was extracted using the PAXgene Blood RNA Isolation Kit (Qiagen, Valencia, CA), following the manufacturer’s instructions. RNA concentration and purity were assessed using the Nanodrop 2000 spectrophotometer (Thermo Scientific, Waltham, MA), evaluating A260/A280 and A260/A230 ratios. The Qubit RNA High Sensitivity (HS) Assay Kit (Thermo Fisher Scientific) was used for accurate quantification. RNA integrity was further validated using the Agilent 2100 Bioanalyzer (Agilent Technologies, Santa Clara, CA), ensuring that all samples met the established thresholds for downstream sequencing applications.

Only RNA samples with optimal integrity and minimal contamination were advanced to library preparation. This strict quality control protocol was crucial in ensuring the reliability of transcriptomic analyses and minimizing technical noise in gene expression profiling.

### Library Preparation and Sequencing

Following the manufacturer’s protocol, RNA samples that passed quality control underwent library preparation using the Universal Plus Total RNA-Seq with NuQuant kit (Tecan Genomics, Redwood City, CA). This protocol employs a stranded, second-strand synthesis approach (fr-second-strand), optimized for the detection of both abundant and low-expression transcripts in whole blood RNA.

RNA integrity was confirmed using the Agilent 2100 Bioanalyzer, and library quality was verified with the Qubit 2.0 Fluorometer (Invitrogen, Carlsbad, CA). To enhance reproducibility, technical replicates were generated for each sample, and only libraries with consistent replicate performance were advanced for sequencing.

Sequencing was performed on the NovaSeq 6000 platform (Illumina, San Diego, CA) using 150-bp paired-end reads, providing high-depth coverage and enabling robust detection of transcript-level expression. Each sample generated an average of ~ 60 million paired-end reads, ensuring sufficient resolution for downstream differential expression analysis and functional enrichment.

This workflow reflects best practices in blood transcriptomics and was designed to optimize sensitivity for detecting gene expression alterations relevant to AD, particularly in genetically diverse and underrepresented populations.

### Bioinformatics Analysis

Raw sequencing reads underwent initial quality control using FastQC (Babraham Bioinformatics) to detect base call errors, adapter contamination, and sequence bias. High-quality reads were then aligned to the GRCh38.p14 human reference genome using the STAR aligner (v2.7.11a), a high-performance tool optimized for spliced read alignment in RNA- Seq datasets [30].

Gene-level quantification was performed using featureCounts (v2.0.0), which reads to genomic features to generate count matrices. Raw counts were normalized using the regularized log (rlog) transformation implemented in DESeq2 (Bioconductor), reducing heteroscedasticity and enabling reliable inter-sample comparison, particularly for low-abundance transcripts.

To reduce noise and increase biological relevance, we applied a minimum expression threshold: genes were required to exhibit more than 0.5 counts per million (CPM) in at least one sample to be retained. Differential expression analysis was conducted using DESeq2, applying a Benjamini–Hochberg false discovery rate (FDR) correction, with a significance threshold set at FDR < 0.05. To ensure biological relevance, we also imposed a log2 fold-change cutoff of ± 1, corresponding to a minimum two-fold difference in expression between groups.

Pathway-level functional enrichment analysis was performed using the Reactome Pathway Database, which employs a hypergeometric test to assess the overrepresentation of differentially expressed genes within curated signaling pathways. To account for multiple testing, *p* values were adjusted using the Benjamini–Hochberg False Discovery Rate (FDR) method, and both raw and adjusted values were retrieved. Pathways with FDR-corrected *q*-values < 0.05 were considered statistically significant.

This multi-step pipeline adheres to best practices in transcriptomic data processing and is particularly well-suited for studies involving ancestrally diverse populations, where technical robustness and noise reduction are essential for detecting biologically meaningful patterns.

### Statistical Analysis

Following the identification of DEGs, we conducted a multivariate analysis to explore the relationships between transcriptomic alterations and neurocognitive outcomes in participants with AD. Specifically, we focused on genes mapped to three major metabolic pathways—protein, lipid, and carbohydrate metabolism, as defined by Reactome functional annotations.

To evaluate the associations between these metabolic signatures and neuropsychological performance, we employed multiple factor analysis (MFA) and variable clustering, both implemented in R (version 4.4.1). These techniques allowed us to integrate heterogeneous datasets, gene expression profiles, and cognitive/clinical measures into a unified analytical framework.

MFA, conducted using the FactoMineR package [31], facilitated the simultaneous analysis of multiple variable groups, identifying shared variance structures between metabolic gene clusters and cognitive domains. We used dimension reduction techniques and visualized associations via biplots to enhance interpretability, highlighting the most influential genes and clinical measures on the principal components.

In parallel, variable clustering, performed using the ClustOfVar package [32], grouped correlated variables based on similarity in their loading patterns. This approach was particularly suited to our analytic goals, as it emphasized variable-level relationships rather than participant-level clustering, relevant in a cohort exclusively composed of AD patients. Clusters were validated using an adapted k-means algorithm, and their stability was assessed through the adjusted Rand index [33], providing a robust measure of internal consistency.

This analytical strategy enabled the identification of transcriptomic profiles most strongly associated with cognitive performance, functional decline, and behavioral symptoms, providing an integrative view of molecular and phenotypic variation in AD.

## Results

### Participant Characteristics and Clinical Profile

A total of 41 individuals were enrolled in the study, including 14 participants with clinically diagnosed AD and 27 CHC. No significant differences were observed between groups in key demographic and anthropometric variables, indicating effective matching and internal validity of group comparisons (Table 1).

**Table 1:** Sociodemographic and functional characteristics

Variable		AD	CHG	*p* value
Age		72.5 (67.25–76.75)	67 (65–74)	0.32^a^
Years of education		8.5 (5–10.75)	10 (5–11)	0.37^a^
Sex	Female	4 (28.57%)	7 (25.93%)	1.00^b^
	Male	10 (71.43%)	20 (74.07%)	1.00^b^
Laterality	Left-handed	2 (14.29%)	1 (3.7%)	0.52^b^
	Right-handed	12 (85.71%)	25 (92.59%)	0.52^b^
	Ambidextrous	0 (0%)	1 (3.7%)	0.52^b^
Marital status	Living together	1 (7.14%)	2 (7.41%)	0.98^b^
	Married	8 (57.14%)	13 (48.15%)	
	Single	3 (21.43%)	7 (25.93%)	
	Widowed	2 (14.29%)	3 (11.11%)	
	Separated	0 (0%)	2 (7.41%)	
SES	1	2 (14.29%)	1 (3.7%)	0.23^a^
	2	3 (21.43%)	3 (11.11%)	
	3	6 (42.86%)	6 (22.22%)	
	4	2 (14.29%)	10 (37.04%)	
	5	1 (7.14%)	6 (22.22%)	
	6	0 (0%)	1 (3.7%)	
BMI		24.18 (22.4–26.99)	25.81 (22.91–27.64)	0.44^a^
Weight		61 (54.25–71.25)	67 (57–77)	0.40^a^
Height		1.6 (1.56–1.64)	1.62 (1.55–1.71)	0.58^a^
Vascular comorbidities				
Arrhythmia	No	13 (92.86%)	26 (96.3%)	1^b^
	Yes	1 (7.14%)	1 (3.7%)	
Cardiac surgery	No	12 (85.71%)	26 (96.3%)	0.26^b^
	Yes	2 (14.29%)	1 (3.7%)	
Diabetes	No	12 (85.71%)	23 (85.19%)	1^b^
	Yes	2 (14.29%)	4 (14.81%)	
Dyslipidemia	No	12 (85.71%)	26 (96.3%)	0.26^b^
	Yes	2 (14.29%)	1 (3.7%)	
Hypertension	No	4 (28.57%)	18 (66.67%)	0.03^b^
	Yes	10 (71.43%)	9 (33.33%)	
Hypothyroidism	No	13 (92.86%)	20 (74.07%)	0.22^b^
	Yes	1 (7.14%)	7 (25.93%)	
FAQ		19.5 (10.25–23.75)	0 (0–2)	< 0.001
FAST		23.5 (14.75–33.5)	2 (1–5)	< 0.001
GDS		2 (1–3)	1 (0.5–3.5)	< 0.001
GAD7		1 (0–2)	2 (0–4.5)	< 0.001
GHQ		9.5 (9–12.5)	9 (6–11.5)	0.47
PSQI		3 (2–5)	3 (2–6.5)	0.44
ISI		2 (0–5.75)	2 (0.5–6)	0.79

Values are presented as median (interquartile range) for continuous and ordinal variables and as frequency (%) for categorical variables. *AD* Alzheimer’s disease, *CHC* cognitively healthy controls, *BMI* body mass index, *FAST* Functional Assessment Staging Tool, *FAQ* Functional Activities Questionnaire, *GDS* Geriatric Depression Scale, *GAD7* Generalized Anxiety Disorder scale, *GHQ* General Health Questionnaire, *PSQI* Pittsburgh Sleep Quality Index, *ISI* Insomnia Severity Index

ᵃ*p* values were calculated using the Mann–Whitney *U* test

ᵇ*p* values were calculated using the chi-square test (*χ*^2^)

The median age was 72.5 years (IQR, 67.25–76.75) in the AD group and 67 years (IQR, 65–74) in the CHC group (*p* = 0.321). Educational attainment was comparable across groups, with a median of 8.5 years (IQR, 5–10.75) in AD versus 10 years (IQR, 5–11) in CHC (*p* = 0.374). Sex distribution was balanced (71.4% male in AD vs. 74.1% in CHC, *p* = 1.00), and similar patterns were observed for body mass index (BMI), weight, and height (all *p* > 0.05). No statistically significant differences emerged in vascular comorbidities, including hypertension, diabetes, dyslipidemia, hypothyroidism, or history of cardiac procedures, except for a higher prevalence of hypertension in the AD group (71.4% vs. 33.3%; *p* = 0.03).

These findings support the clinical comparability of groups, minimizing confounding effects related to demographic or vascular risk factors in downstream transcriptomic and cognitive analyses.

### Functional and Neurocognitive Performance

As expected, participants with AD demonstrated substantial functional impairment relative to CHC, as reflected by elevated scores on the FAQ (median = 19.5 [IQR, 10.25–23.75] vs. 0 [IQR, 0–2], *p* < 0.001) and the FAST (23.5 [IQR, 14.75–33.5] vs. 2 [IQR, 1–5], *p* < 0.001).

Cognitive testing revealed pronounced deficits in the AD group across all domains (see Table 2). Global cognitive function, assessed by the ACE-R, was markedly reduced (median = 55.5 [IQR, 48.5–67] vs. 95 [IQR, 92.5–98], *p* < 0.001), as was performance on the MoCA (9 [IQR, 7–11] vs. 20 [IQR, 18.5–21], *p* < 0.001). Executive functioning, measured via the INECO Frontal Screening (IFS), was also significantly impaired in AD (3.61 [IQR, –0.56–8.16] vs. 16.19 [IQR, 15.12–18.19], *p* < 0.001).

**Table 2:** Neuropsychological assessment

Variable	AD	CHG	*p* value
ACE-R	55.5 (48.5–67)	95 (92.5–98)	< 0.001
MoCA	9 (7–11)	20 (18.5–21)	< 0.001
IFS	3.61 (−0.56–8.16)	16.19 (15.12–18.19)	< 0.001
UDS-NACC Composite memory	2 (0.5–3.75)	40 (36–47)	< 0.001
UDS-NACC Composite language	48.5 (37.5–65.25)	93 (88.5–101.5)	< 0.001
UDS-NACC Composite visuospatial	11 (8–15)	17 (16–17)	< 0.001
MINISEA	14.71 (9.21–19.81)	25.77 (23.84–26.79)	< 0.001

Values are expressed as median (interquartile range)

*AD* Alzheimer’s disease, *CHC* cognitively healthy controls, *MoCA* Montreal Cognitive Assessment, *ACE-R* Addenbrooke’s Cognitive Examination–Revised, *IFS* INECO Frontal Screening, *UDS-NACC* Uniform Data Set from the National Alzheimer’s Coordinating Center, *MINI-SEA* Mini Social Cognition and Emotional Assessment

*p* values were calculated using the Mann–Whitney *U* test

Composite scores from the UDS-NACC battery further confirmed deficits in episodic memory (2 [IQR, 0.5–3.75] vs. 40 [IQR, 36–47]); language (48.5 [IQR, 37.5–65.25] vs. 93 [IQR, 88.5–101.5]); and visuospatial abilities (11 [IQR, 8–15] vs. 17 [IQR, 16–17]); all comparisons *p* < 0.001. Social cognition, assessed via the Mini-SEA, was likewise compromised in the AD group (14.71 [IQR, 9.21–19.81] vs. 25.77 [IQR, 23.84–26.79], *p* < 0.001).

In contrast, affective symptoms and sleep-related disturbances, evaluated via GDS, GAD-7, GHQ-12, PSQI, and ISI, did not differ significantly between groups, suggesting that neurocognitive and functional impairments were not confounded by concurrent mood or sleep disturbances in this cohort.

### Differential Gene Expression Analysis

**Fig. 1:**
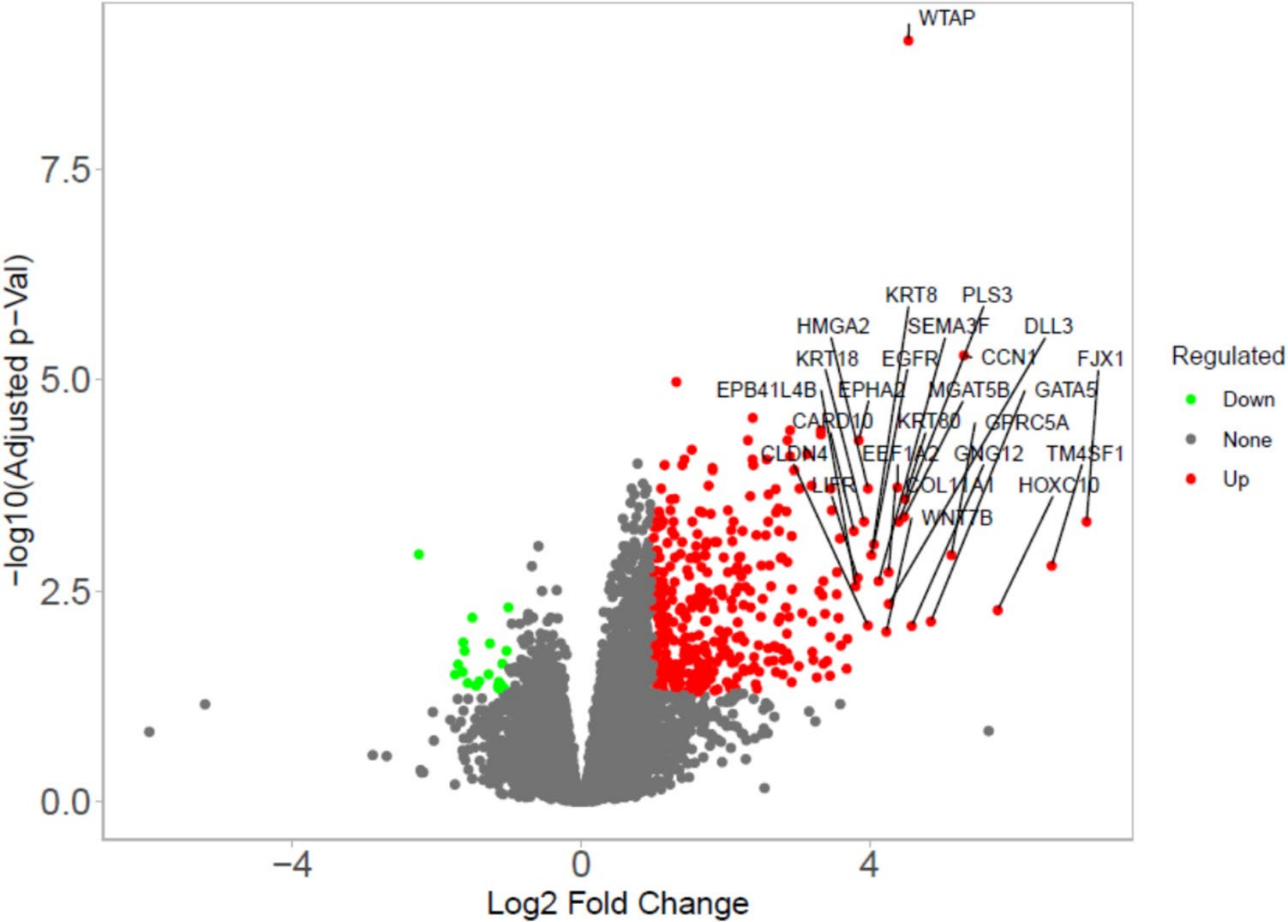
Volcano plot of the DEGs. The *x*-axis represents log2 FC, which indicates the mean expression level for each gene. The *y*-axis represents the adjusted *p* value (-log 10). Each dot represents one gene

Transcriptomic profiling was performed on high-quality RNA extracted from peripheral blood samples of 41 participants. After preprocessing and filtering, a total of 38,293 genes were retained for downstream analysis. Using a conservative log₂ fold-change threshold of ± 1 and an FDR < 0.05, we identified 399 DEGs between the AD and CHC groups. Among these, 378 genes were upregulated and 21 were downregulated in the AD group (Fig. 1).

A volcano plot revealed a clear transcriptional signature differentiating AD from controls, with several high-impact genes exceeding both statistical and biological significance thresholds. Notably, APOE (log₂FC = 2.95), MMP2 (log₂FC = 3.34), PPARG (log₂FC = 2.00), and TUBB3 (log₂FC = 2.92) were prominently upregulated in the AD cohort.

### Functional Enrichment Analysis of DEGs

Functional annotation of DEGs using the Reactome Pathway Database revealed significant dysregulation across several core biological systems. A total of 61 DEGs were mapped to the Metabolism of Proteins pathway (R-HSA-392499), corresponding to an entity ratio of 0.15, indicating that approximately 15% of the genes involved in this pathway were altered in AD participants (Table 3).

**Table 3:** Enriched pathways derived from DEGs with significant alterations in metabolic and neuronal system

Pathway name	Entities found	Entities total	Entities ratio
1. Metabolism	49	3752	0.24
1.1 Metabolism of carbohydrates	6	454	0.029
1.2 Metabolism of lipids	11	1500	0.096
2. Metabolism of proteins	61	2353	0.15
2.1 Translation	19	339	0.022
2.2 Post-translational protein modification	33	1661	0.106
3. Neuronal system	10	490	0.031
3.1 Transmission across chemical synapses	8	344	0.022

**Fig. 2:**
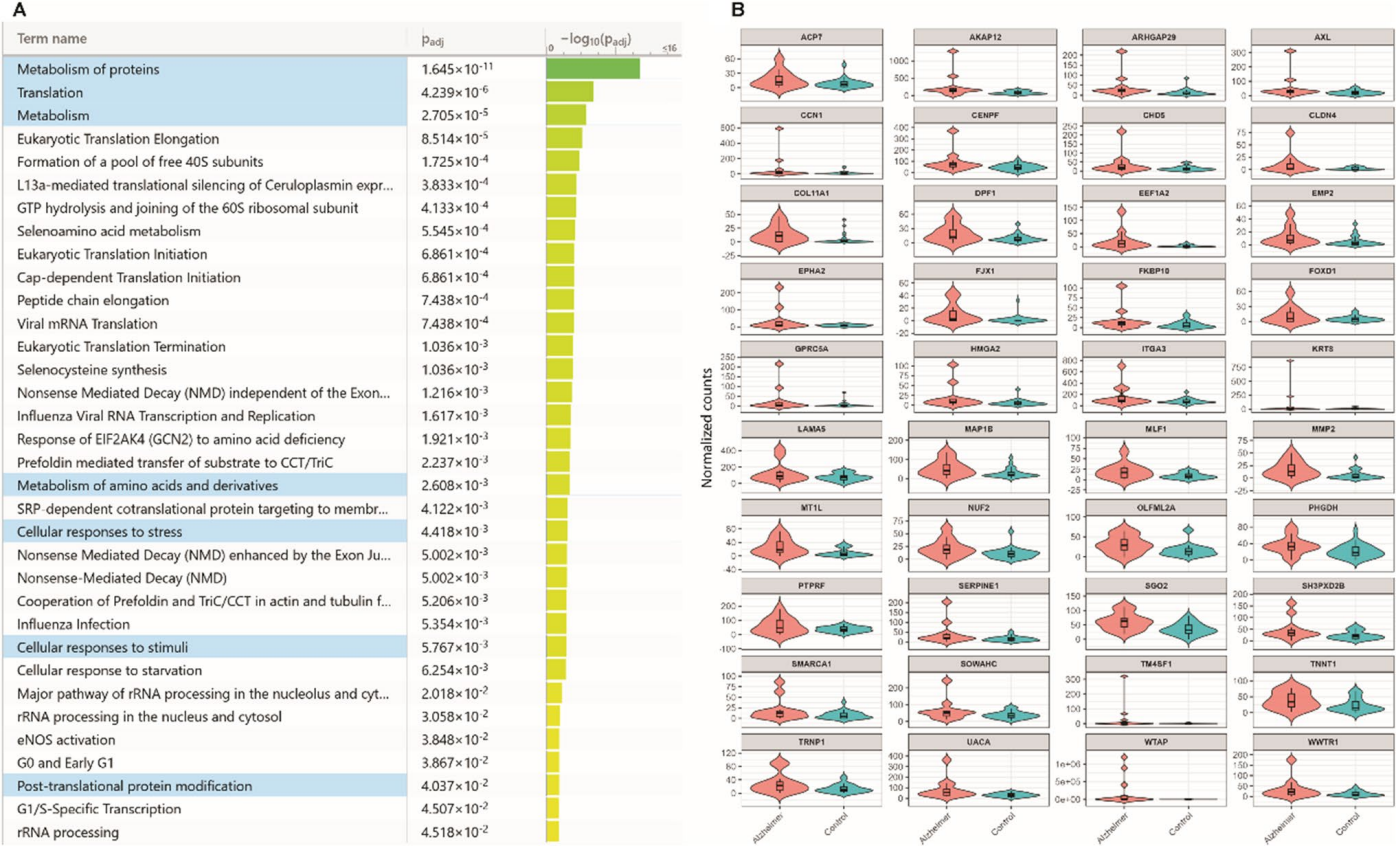
Reactome pathway enrichment and expression distribution of representative genes. **A** Enrichment analysis of Reactome pathways and differential gene expression in Alzheimer’s disease. **B** Violin plots showing the expression patterns of the 40 core genes associated with the significantly enriched Reactome pathways. AD (pink) and control (blue) groups

Based on the Reactome pathway enrichment analysis, we identified a robust set of signaling pathways significantly associated with Alzheimer’s disease, even after adjustment for multiple testing (Fig. 2A). The most enriched categories included protein metabolism (*P*adj = 1.645 × 10⁻^11^), translation (*P*adj = 4.239 × 10⁻^6^), and cellular responses to stress (*P*adj = 4.418 × 10⁻^3^), highlighting the central role of dysregulated protein homeostasis and stress adaptation mechanisms in the disease. Complementary inspection of the 40 prioritized genes associated with these pathways revealed distinct expression profiles between Alzheimer’s and control groups (Fig. 2B). Notably, genes such as LAMA5, MAP1B, SERPINE1, and COL11A1 were consistently overexpressed in Alzheimer’s samples. In contrast, others, such as WTAP and KRT8, showed more heterogeneous distributions.

In addition to metabolic disruption, functional enrichment analysis highlighted significant alterations in the Transmission across Chemical Synapses pathway (Reactome ID: R-HSA-112315), a core neuronal signaling system. Key DEGs implicated in this pathway included TUBB3, CACNA2D1, GABBR2, GRIN2A, GNG12, and CACNG4, all involved in synaptic plasticity, neurotransmitter release, and calcium-mediated signaling. These findings suggest that peripheral blood may capture transcriptomic footprints of disrupted synaptic function in AD. Other enriched pathways encompassed lipid and carbohydrate metabolism, mitochondrial biogenesis, and muscle contraction, with DEGs such as ATP8, MAPK11, ANXA2, SDC4, TPM2, and TNNT1 pointing to broader metabolic and structural impairments.

The pathway enrichment analysis, which was based on the differential expression results, highlighted dysregulation in “Metabolism of RNA,” “Signal Transduction,” and “Metabolism of proteins.” Among the genes contributing to these pathway-level findings were several with strong upregulation, including TM4SF1 (log₂FC = 6.39), GPRC5A (5.41), FJX1 (4.66), EPHA2 (4.50), and CCN1 (4.41). Furthermore, we observed relevant increases in other genes such as COL11A1 (3.27), KRT8 (2.92), HMGA2 (2.88), SERPINE1 (2.72), and ARHGAP29 (2.70), which are known to play roles in extracellular matrix remodeling, cytoskeletal organization, and cellular stress response.

### Multivariate Integration of Molecular and Neurocognitive Data

To investigate the multidimensional associations between DEGs and cognitive, functional, and clinical profiles in AD, we conducted a multiple factor analysis (MFA). This technique integrates transcriptomic data with behavioral assessments by reducing complex variable sets into orthogonal dimensions of shared variance.

**Fig. 3:**
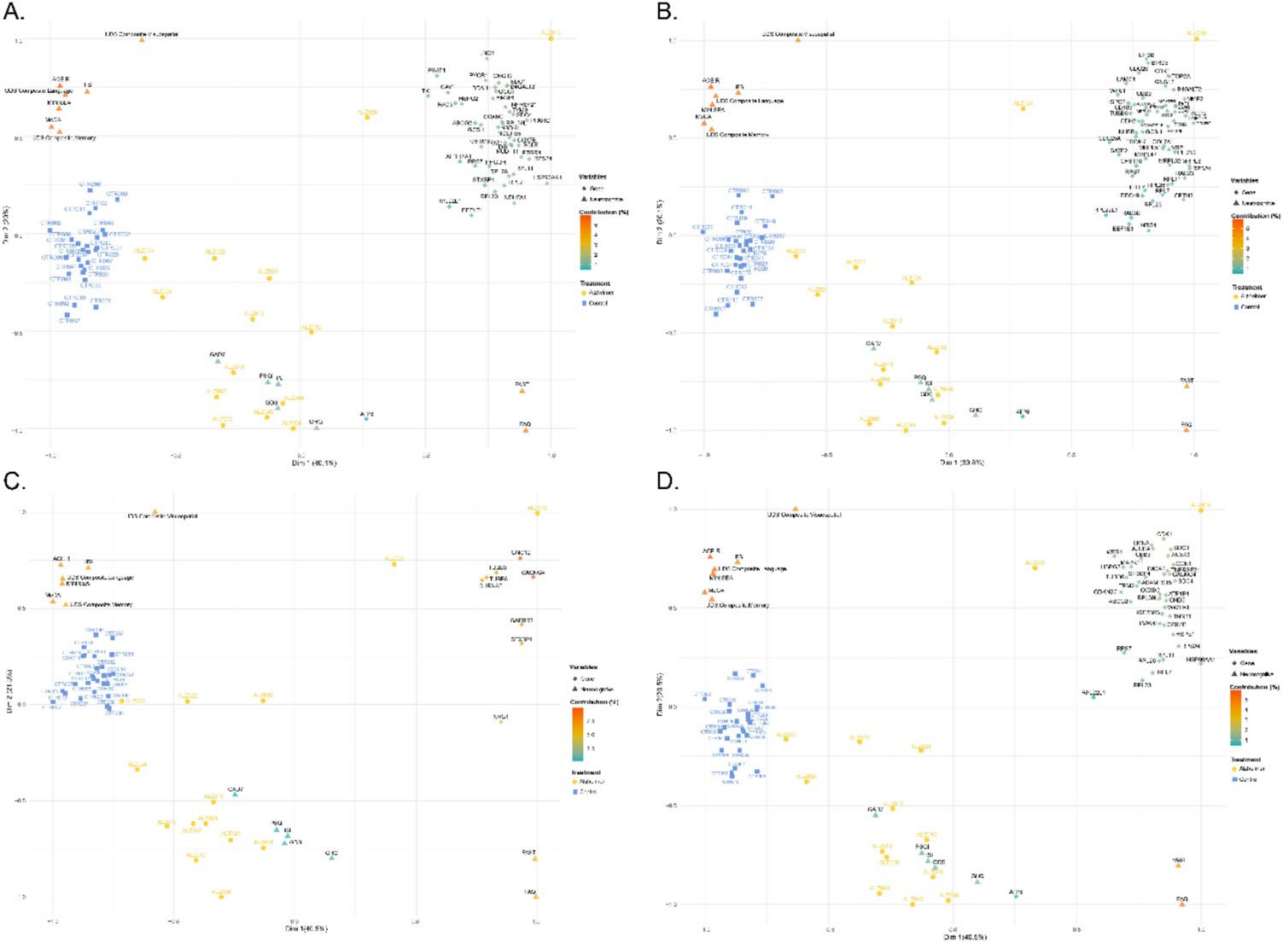
Multiple factor analysis (MFA) integrating differentially expressed genes (DEGs) and neurocognitive, functional, and clinical measures in Alzheimer’s disease. **A** Metabolism pathway (carbohydrates and lipids). **B** Metabolism of proteins pathway. **C** Transmission across chemical synapses pathway. **D** Diseases of metabolism, cellular responses to stress, insulin-like growth factor-2 mRNA binding proteins (IGF2BPs/IMPs/VICKZs) bind RNA, mitochondrial biogenesis, muscle contraction

In our multivariate analysis, Alzheimer’s patients (yellow) were separated from controls (blue) along Dimension 1 (~ 40% of explained variance), while Dimension 2 (~ 20.0%) captured additional variability associated with neurocognitive and clinical performance (Fig. 3A–D). The Permanova confirmed that group differences were statistically significant (*F* ~ 9, *R*^2^ = ~ 0.2, *p* = 0.001) (Supplementary Table 1), indicating that approximately 20% of the variance in the multivariate model is attributable to disease status. Across the four MFA panels, Dimension 1 was consistently driven by global cognition and memory, with MoCA (6.3–6.4%), ACE-R (6.0%), and UDS Composite Memory (5.9%) as top contributors. Dimension 2 was dominated by visuo-executive functions, led by UDS composite visuospatial (≈9–10%), followed by ACE-R (≈5.9%) and IFS (≈5.5%), together capturing heterogeneity in cognitive and functional status within the AD cohort. On the molecular side, distinct sets of differentially expressed genes contributed to the separation across pathways: ribosomal proteins (RPL22L1, RPL11, RPS24); mitochondrial regulators (ATP8, NDUFA1, COX7B); and synaptic genes (STXBP1, TUBB3, CACNG4, GABBR2) showed strong loadings, highlighting dysregulation in proteostasis, metabolism, and synaptic signaling. In particular, synaptic transmission genes aligned with Dimension 1, underscoring their role in cognitive decline, while mitochondrial and metabolic regulators were more strongly associated with Dimension 2, linking them to variability in functional outcomes (FAQ, FAST) (Supplementary Table 2). Altogether, these results suggest that Alzheimer’s disease is characterized by coordinated clinical and transcriptomic axes of variability, reflecting the convergence of cognitive decline, functional impairment, and molecular dysregulation in energy and synaptic pathways.

## Discussion

This exploratory transcriptomic study aimed to identify DEGs in peripheral blood samples from patients with AD and to explore the biological pathways and phenotypic correlations underlying these molecular alterations. Using high-throughput RNA sequencing and integrative multivariate analyses, we demonstrate that AD is characterized by systemic transcriptional dysregulation, particularly in metabolism, synaptic transmission, and mitochondrial function pathways. These findings reinforce the emerging view that peripheral molecular signatures may serve as accessible and biologically meaningful markers of neurodegeneration. The use of peripheral blood is especially relevant in resource-limited settings, where cerebrospinal fluid biomarkers and advanced neuroimaging are often not feasible [4, 34].

This work addresses a persistent gap in AD research regarding the systematic exclusion of ancestrally diverse and socioeconomically underrepresented populations from transcriptomic and biomarker discovery studies [8, 13]. By focusing on a cohort from Valle del Cauca, Colombia, a region marked by complex admixture of Amerindian, African, and European ancestry [19, 20], we contribute to a more inclusive molecular characterization of AD. This is particularly relevant given increasing evidence that genetic ancestry influences gene expression, immune response, and disease susceptibility [16, 18].

Among the 399 DEGs identified, several mapped biologically plausible mechanisms previously linked to AD. Notably, upregulation of TUBB3, GABBR2, CACNA2D1, and GRIN2A implicates synaptic dysregulation, glutamatergic signaling, and calcium channel alterations, processes critical for plasticity and neuronal communication [35, 36]. These genes have also been implicated in populations with metabolic comorbidities, reinforcing the convergence between neurodegeneration and insulin resistance.

Indeed, prior studies suggest that metabolic dysfunction exacerbates amyloid aggregation, neuroinflammation, and vascular injury, all of which contribute to cognitive decline in AD [37]. Consistent with this evidence, our findings support the role of insulin resistance, mitochondrial dysfunction, and altered protein metabolism in AD progression. Impaired insulin signaling can trigger tau hyperphosphorylation, amyloid-beta accumulation, and synaptic failure via activation of the JNK pathway and serine phosphorylation of insulin receptor substrates (IRS-1/2), ultimately disrupting neuronal glucose metabolism [38–40].

Mitochondrial dysfunction is a downstream consequence of this metabolic dysregulation, resulting in decreased ATP production and increased oxidative stress. The resultant redox imbalance promotes the generation of reactive oxygen species (ROS), which damage mitochondrial DNA, impair neuronal membranes, and accelerate the aggregation of amyloid-beta and tau [41, 42]. These mechanisms have also been reported in diabetic individuals with microvascular brain injury, suggesting that metabolic and neurodegenerative processes share overlapping biological substrates [43].

The integration of transcriptomic and clinical data revealed specific transcriptional profiles associated with cognitive and functional decline. Genes such as ATP8 and FOLR3 correlated with lower global cognition, poor sleep quality, and emotional distress. Conversely, synaptic transmission–related genes such as STXBP1, GABBR2, and GNG12 displayed inverse relationships with depressive symptoms, raising hypotheses regarding the neurobiological dissociation between affective and cognitive trajectories in late-stage AD.

From a methodological standpoint, this study also contributes to the field by combining rlog-normalized gene expression, pathway-level enrichment, and multiple factor analysis to uncover multivariate relationships between molecular and phenotypic AD dimensions. Few transcriptomic studies in Latin America have employed this level of analytical integration, reinforcing the potential of such approaches for biomarker discovery in ancestrally diverse populations.

Multiple studies from different countries that used RNA-seq data from other patients with AD were revised to have better understanding of the influence of ancestry and geographical location on gene expression signatures in AD and compare their results with those obtained in this study with a Latin American cohort. The study by Shigemizu et al. [12], conducted on a Japanese population, identified eight genes, RPS24, RPL7, RPL5, RPS5, RPS12, RPS3A, RPS6, and RPL23A, which are also present in our list of DEGs. The discovery of these ribosomal protein genes may suggest a conserved role in AD pathology across different ancestral backgrounds, though their specific function and expression patterns would require further validation. Other studies performed in Taiwan [44] and the USA [45], also identified DEGs in common with our list, CKS2 and HSD17B10 respectively. Also, Sood et al. [46] conducted an RNA-seq study with population from the UK and found four DEGs that matched those found in our study, POLR2K, COX6C, PLK2, and MAP1B.

However, we did not find other DEGs that overlap with the studies from Iran [47], the USA [48], Spain [49], or China [50]. These results might suggest that ancestry differences substantially affect the specific genes influencing AD, potentially due to genetic diversity, environmental factors, or lifestyle differences that modulate gene expression and disease mechanisms. For clarity, we note that genetic ancestry was not specified in most of the comparator RNA-seq studies; therefore, we retained the population descriptors reported by the original authors, by country or region, to avoid inferring ancestry from geography.

This systematic comparison highlights that, while some core pathological processes, such as ribosomal function or immune response, may be common in AD, the specific DEGs can vary considerably across different ancestral populations. Therefore, our list of 399 DEGs identified in the analyzed samples, although robust within our cohort, may represent a population-specific expression profile. Future research should prioritize multi-ancestral cohorts to distinguish globally conserved AD biomarkers from those specific to certain populations, which is essential for developing equitable and effective diagnostic tools worldwide.

## Limitations

The interpretation of these findings requires consideration of certain limitations. The sample size, while comparable to other exploratory transcriptomic studies [12, 51], restricts generalizability and statistical power. Additionally, because covariates were managed a priori through case–control matching rather than included in the differential expression model, residual confounding by unmeasured variables such as sex, age, or comorbidities, cannot be entirely excluded. The use of whole blood, a heterogeneous tissue, may mask cell–type–specific signals, and the cross-sectional design precludes causal inference. The absence of an independent replication cohort also underscores broader structural inequities in genomic research across LMIC.

Although peripheral blood was collected for transcriptomic profiling and APOE genotyping, these exploratory molecular measures were not used as diagnostic criteria. Consequently, our study cannot fully exclude the presence of co-pathologies such as vascular, Lewy body, or frontotemporal dementia, or the possibility of diagnostic misclassification. The inclusion of 3 T MRI helped minimize this risk by excluding major cerebrovascular or structural lesions; however, subclinical or mixed pathologies remain possible. Likewise, cognitively healthy controls may include individuals in a preclinical stage of AD, such as amyloid-positive but asymptomatic participants. These limitations are inherent to the absence of CSF- or PET-based biomarkers, but they reflect the realities of recruitment in low- and middle-income settings.

Despite these caveats, this study offers critical exploratory evidence that transcriptomic biomarkers from underrepresented, ancestrally diverse populations can inform the molecular understanding of AD and support the development of more inclusive and equitable diagnostic tools. In the context of global precision medicine, this work represents a crucial step toward closing the equity gap in biomarker science and advancing translational neurogenomics in regions that have been historically marginalized from genomic innovation.

## Conclusion

This study provides exploratory evidence that peripheral transcriptomic profiles capture biologically and clinically meaningful signatures of AD in an ancestrally diverse and historically underrepresented population. Through RNA sequencing of whole blood and integration with neurocognitive assessments, we identified 399 DEGs enriched in pathways related to synaptic function, mitochondrial metabolism, and cellular stress response, core systems implicated in AD pathophysiology.

This work addresses the persistent gap in the representation of non-European populations in neurogenic research by focusing on a Colombian cohort with high genetic admixture. The findings underscore the potential utility of blood-based gene expression as a minimally invasive biomarker source, particularly in resource-constrained settings where access to cerebrospinal fluid or advanced imaging is limited.

Methodologically, the integration of transcriptomic and clinical data using multiple factor analysis revealed discrete molecular clusters associated with cognitive decline, functional impairment, and neuropsychiatric symptoms. These results provide a framework for future research to develop systems-level biomarkers that can capture the heterogeneity of AD presentation. However, the cross-sectional design, modest sample size, and absence of replication constrain the generalizability of the findings. This study should be viewed as a proof-of-concept, encouraging validation in larger, longitudinal, and multicenter cohorts across diverse ancestral backgrounds.

Ultimately, this work supports the growing recognition that precision medicine in AD must include underrepresented populations. Expanding molecular research to such communities is not only a scientific imperative, but also a path toward equity in the future of neurodegenerative diagnostics and therapeutics.

## Supplementary Information

Below is the link to the electronic supplementary material.

ESM 1 (11.0 KB XLSX)

ESM 2 (19.0 KB XLSX)

## Data Availability

The data that support the findings of this study are available from the corresponding author, upon reasonable request.

## Abbreviations

ACE-R: Addenbrooke’s Cognitive Examination-Revised
AD: Alzheimer’s disease
BBB: Blood-brain barrier
BMI: Body mass index
CDR: Clinical dementia rating
CHC: Cognitive healthy controls
DEGs: Differentially-expressed genes
FAQ: Functional Activities Questionnaire
FAST: Functional Assessment Staging Tool
FDR: False Discovery Rate
GHQ-12: General Health Questionnaire
ISI: Insomnia Severity Index
NINCDS–ADRDA: National Institute of Neurological and Communicative Diseases and Stroke–Alzheimer’s Disease and Related Disorders Association
MFA: Multiple factor analysis
MMSE: Mini-Mental State Examination
MoCA: Montreal Cognitive Assessment
PCA: Principal component analysis
PSQI: Pittsburgh Sleep Quality Index
